# Supplementary material for: Convergent evolution of Y chromosome gene content in flies
Source: Nat Commun. 2017 Oct 4;8:785. doi: 10.1038/s41467-017-00653-x (PMC5627270; doi:10.1038/s41467-017-00653-x)
Supplement: Supplementary file 1 — Supplementary Information [file 41467_2017_653_MOESM1_ESM.pdf]

File name: Supplementary Information
Description: Supplementary Figures, Supplementary Tables and Supplementary References

File name: Supplementary Data 1
Description: Sequences of all assembled putative Y-linked transcripts in fasta format.

File name: Supplementary Data 2
Description: Sequences of paralogs of putative Y-linked transcripts in fasta format.

File name: Peer Review File

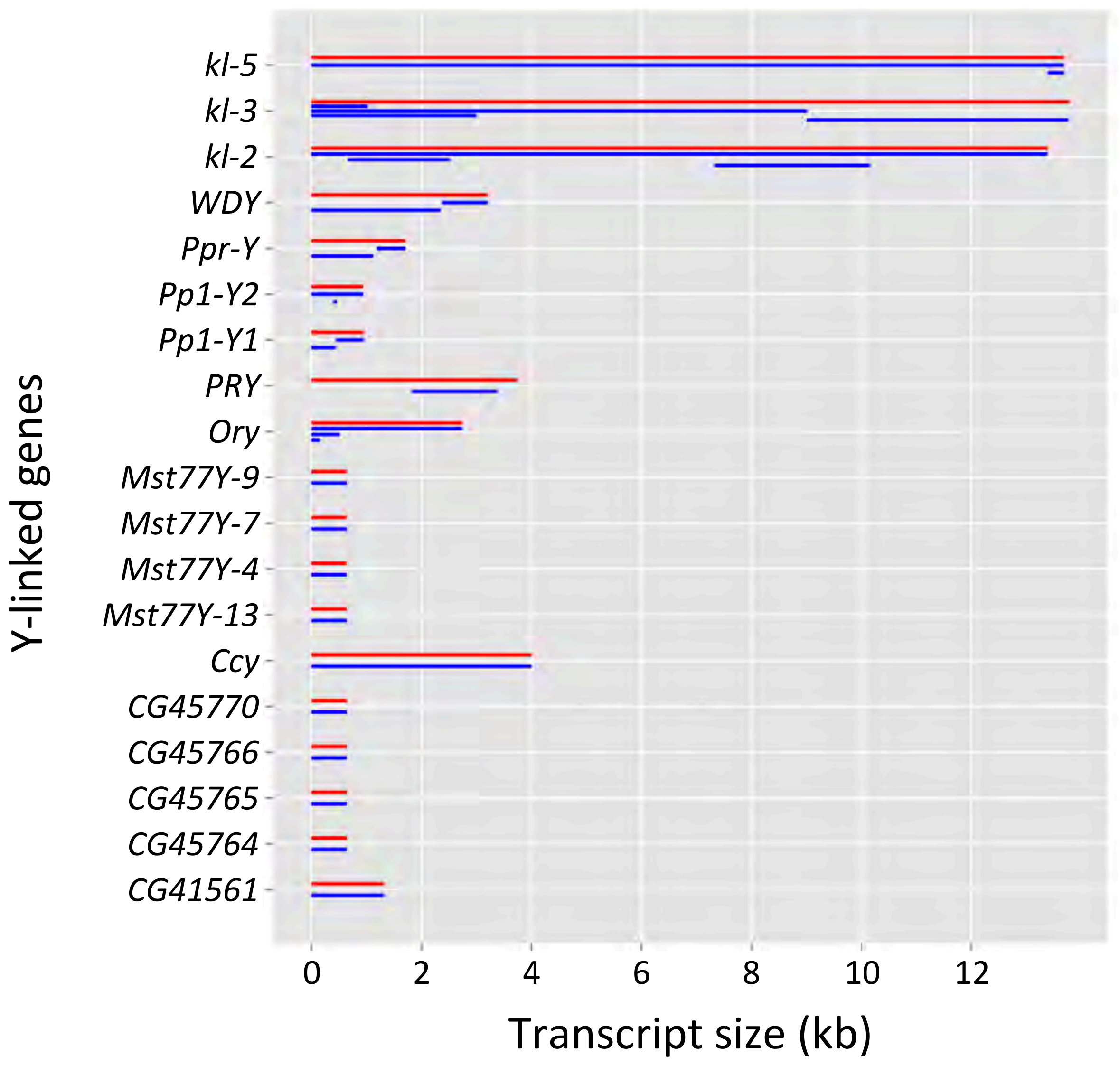

Supplementary Figure 1. Validation of bioinformatics pipeline to infer Y-linked transcripts in *D. melanogaster.* All annotated transcripts of the *D. melanogaster* Y chromosome are shown as red lines. Blue lines indicate the alignment of assembled transcripts using our subtraction pipeline (from Fig. 1) against known *D. melanogaster* Y genes. The majority of genes on the *D. melanogaster* Y chromosome are covered by a single transcript assembled by our pipeline. For some genes, multiple isoforms were assembled by our pipeline (overlapping blue lines).

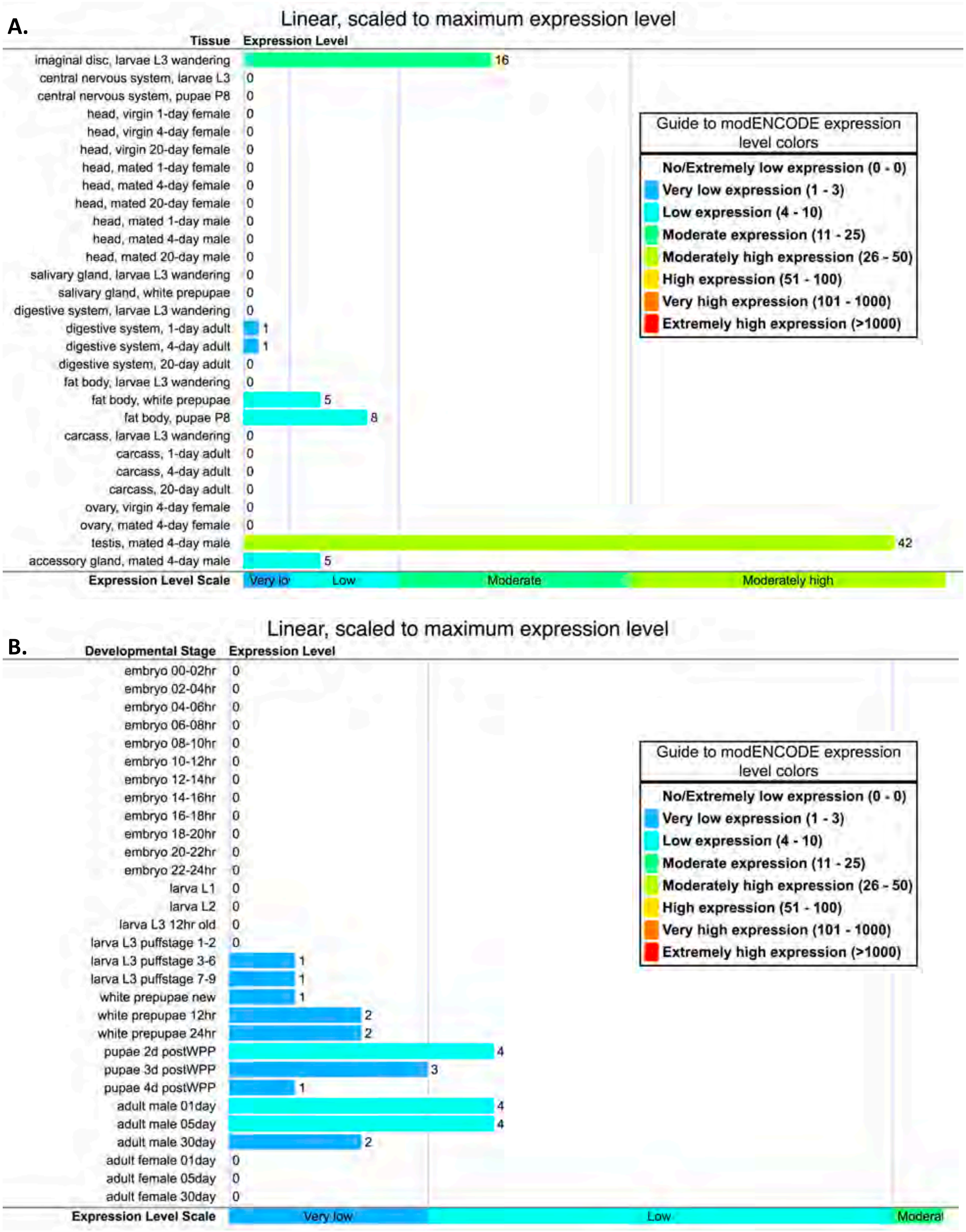

Supplementary Figure 2. Expression profile of the new Y-linked gene *CG41561*. (**a**) Tissue expression and (**b**) developmental stage expression for *CG41561*/transcript TR3794 (images taken from flybase).

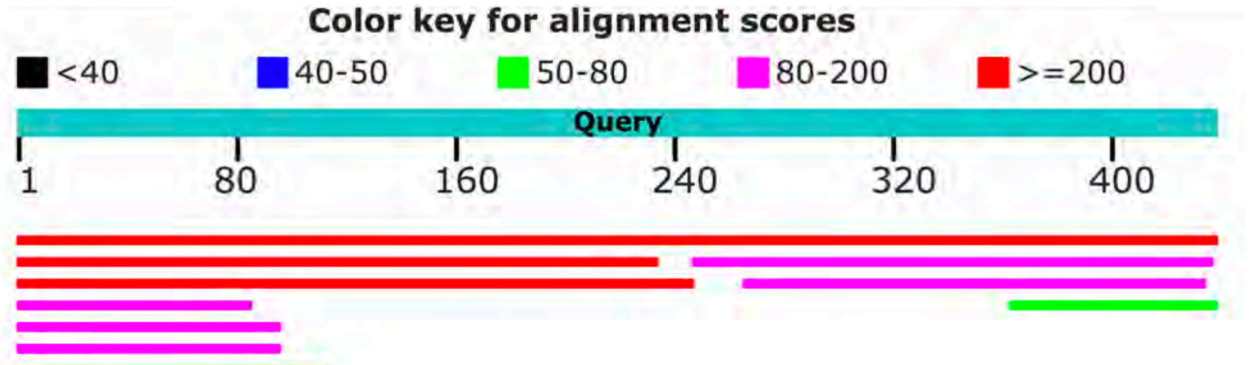

Description	Max score	Total score	Query cover	E value	Ident	Accession
uncharacterized protein Dmel_CG41561 [Drosophila melanogaster]	897	897	100%	0.0	100%	NP_001104078.2
MIP14882p [Drosophila melanogaster]	483	483	53%	3e-169	100%	ADB91442.1
uncharacterized protein Dere_GG26511 [Drosophila erecta]	305	305	56%	2e-98	62%	XP_015008604.1
uncharacterized protein Dere_GG27111 [Drosophila erecta]	193	193	43%	1e-55	58%	XP_015008591.1
uncharacterized protein Dyak_GE28614 [Drosophila yakuba]	161	161	38%	2e-44	53%	XP_015045276.1
uncharacterized protein LOC110187192 [Drosophila serrata]	92.0	92.0	19%	4e-17	54%	XP_020812251.1
uncharacterized protein Dana_GF17090 [Drosophila ananassae]	85.1	85.1	22%	5e-14	49%	XP_001953716.2

Supplementary Figure 3. Blastp results for *CG41561*.

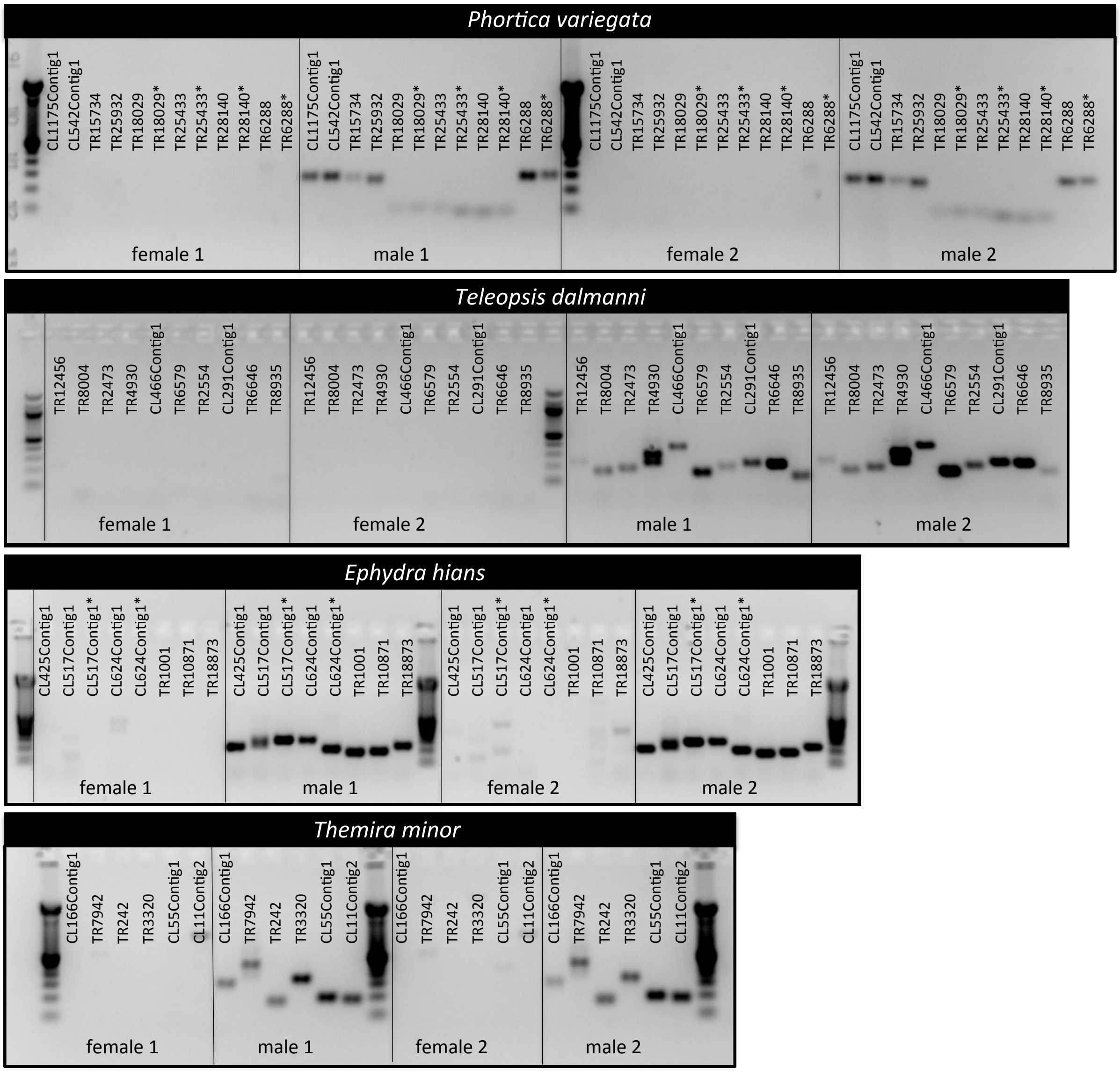

Supplementary Figure 4. PCR confirmation of Y-linked transcripts in *Phortica variegata, Teleopsis dalmanni*, *Ephydra hians* and *Themira minor*. Y-linked transcripts amplify with male genomic DNA, but not with female genomic DNA.

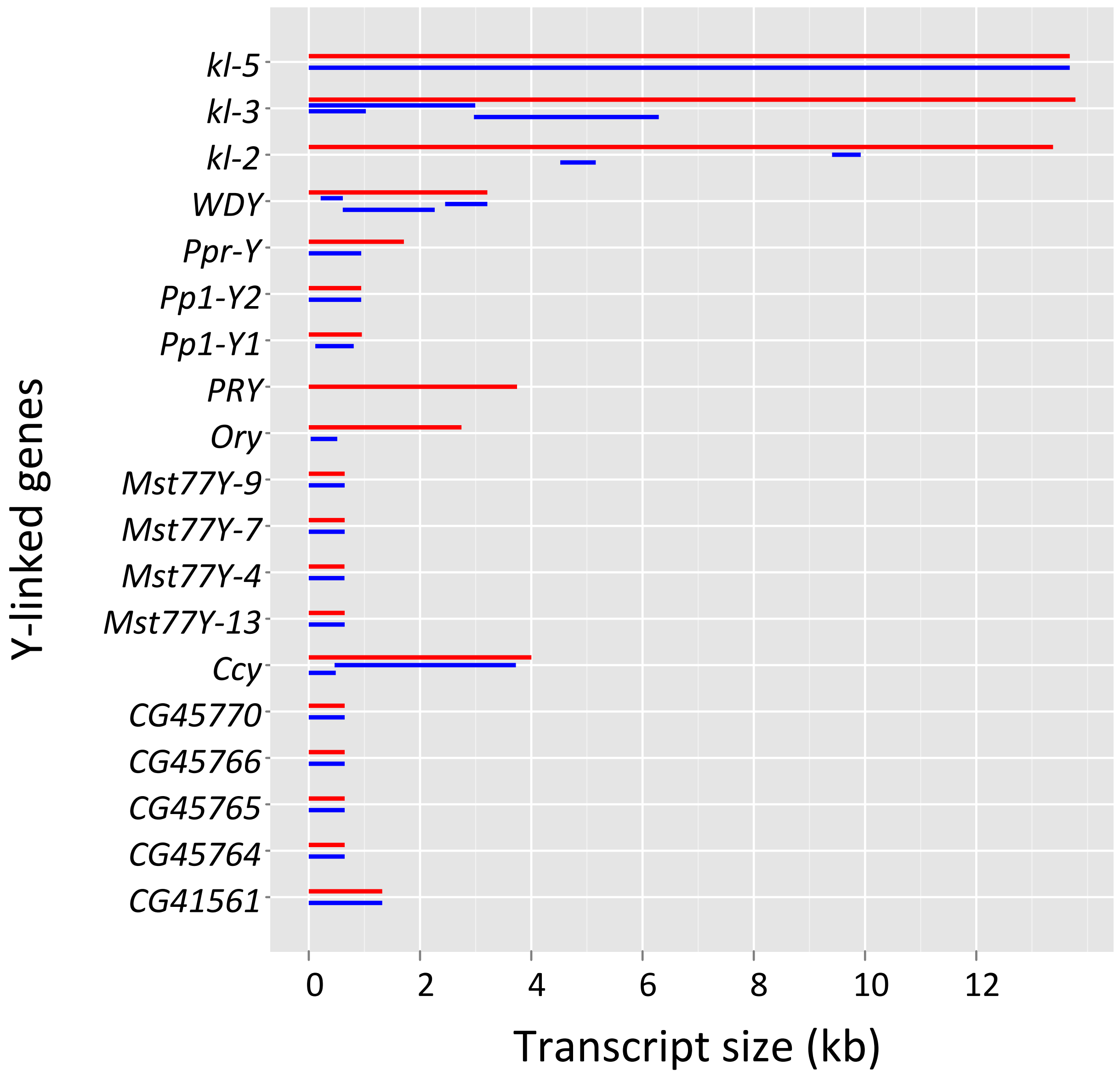

Supplementary Figure 5. Validation of our pipeline with subsampled *D. melanogaster* data. Same as Supplementary Figure 1, but we subsampled our *D. melanogaster* data to match read counts with the species for which we have the lowest number of genomic read pairs (*Mayetiola destructor*).

```
Query: A.gambiae-TR578|c0_g1_i1 len=681 path=[659:0-680] [-1, 659, -2]
> X
dna:chromosome chromosome:AgamP4:X:1:24393108:1
Length = 24393108

Score = 632
Expect = 2e-161
Identity = 82.7648%
Strand = Plus/Minus
Aln Length = 553

Frame: 1 / -1

isTagged: , hitName: X
Query  129       TGATGGAGCGTGCGGAGAACATCACGCAGCGCAGGAAGCGTAGCTGGCTCCGGTTTCGTT  188
                 ||| || ||||||| ||  || |||||| | |||||||| |||||| ||||||||||| |
Sbjct  10685285  TGACGGTGCGTGCGCAGGGCAACACGCACCACAGGAAGCATAGCTGACTCCGGTTTCGAT  10685226

Query  189       TGATAGCGGCACAAATACTGTTCACCGCCACCCACCTTGGTGTTCTGCACGGCATCTCGA  248
                 |||||||||||||||||||||| | ||||||||   |||| |||||   |||||||||||
Sbjct  10685225  TGATAGCGGCACAAATACTGTTAATCGCCACCCTGTTTGGCGTTCTTATCGGCATCTCGA  10685166

Query  249       TCTTGAGGTATTTGAGAGCATGCAGGTCTGCGAAGGCCGGCCAATTGACGGCCACCGAAC  308
                 ||||||||||||| ||||| |||                  |||||||| ||||||||||
Sbjct  10685165  TCTTGAGGTATTTAAGAGCCTGC------------------CAATTGACCGCCACCGAAC  10685088

Query  309       CTACCGTAAA-GTTTTCCAGCCGCGGCAGCTTAATGTTCGCGAACGAAAGGGGCGTAGTG  367
                 | ||||| |  |||||||||||||||||  | ||||| |||||||||| ||| ||| |||
Sbjct  10685087  CCACCGTCAGCGTTTTCCAGCCGCGGCAATTCAATGTCCGCGAACGAATGGGACGTGGTG  10685028

Query  368       CTGTACAAAATTTTCAACTGGCGCAAGCCGGGCGGGTTCGCTTACAAGCGCAATGGGTTG  427
                 |||  ||||||||||| |||||||||||||| ||| ||||||  |||||||||||||||
Sbjct  10685027  CTGAGCAAAATTTTCAGCTGGCGCAAGCCGGACGGATTCGCTAGCAAGCGCAATGGGTTT  10684968

Query  428       CCGCTGCACTCTGCCACGTTAAATATGTACAAGTCCTCCAGCAGGACACACGATTGCCCG  487
                 ||||||||||||||||||||||| |||| ||||||||||||| |||||||||||| ||||
Sbjct  10684967  CCGCTGCACTCTGCCACGTTAAAGATGTGCAAGTCCTCCAGCTGGACACACGATTTCCCG  10684908

Query  488       ATGGCCTGCAGTACG---TCCAACACGGTATAAAATTGCAGGCGCAGCTTCTTTGCTGCT  544
                 |||||||||||||||   ||| || |||||||||||||||||||||||||||||   |||
Sbjct  10684907  ATGGCCTGCAGTACGCTTTCCGACGCGGTATAAAATTGCAGGCGCAGCTTCTTT--AGCT  10684846

Query  545       GACTCAACTGCCGGGaaaaaaaCAGTACCTTGTGCAGTACCTTGCTCAACTGTAGGTGTT  604
                   ||||||||||||   |||||||||||||| ||  |||| |    ||||    ||||||
Sbjct  10684845  AGCTCAACTGCCGG---AAAAACAGTACCTTTTGTTGTACAT---CCAAC----GGTGTT  10684776

Query  605       TTAGTGAACGTTGGCTTCAACTCGACCTCTATTTCGTGGCTTATGATCAGCTGCTCGAGC  664
                   ||||||||||||| ||||||| |||||  |||||||||||||||||||||||| ||||
Sbjct  10684775  GGAGTGAACGTTGGCATCAACTCAACCTCCGTTTCGTGGCTTATGATCAGCTGCTGGAGC  10684716

Query  665       TGCTTCAGCTGCCAGTA                                            681
                 |||||||||||||||||
Sbjct  10684715  TGCTTCAGCTGCCAGTA                                            10684699
```

Supplementary Figure 6. Alignment between the putative Y-linked transcript *A. gambiae* TR578 and an X-linked region in *A. gambiae*. The transcript TR578 is homologous to parts of the previously described *YG1*/*YG2* genes.

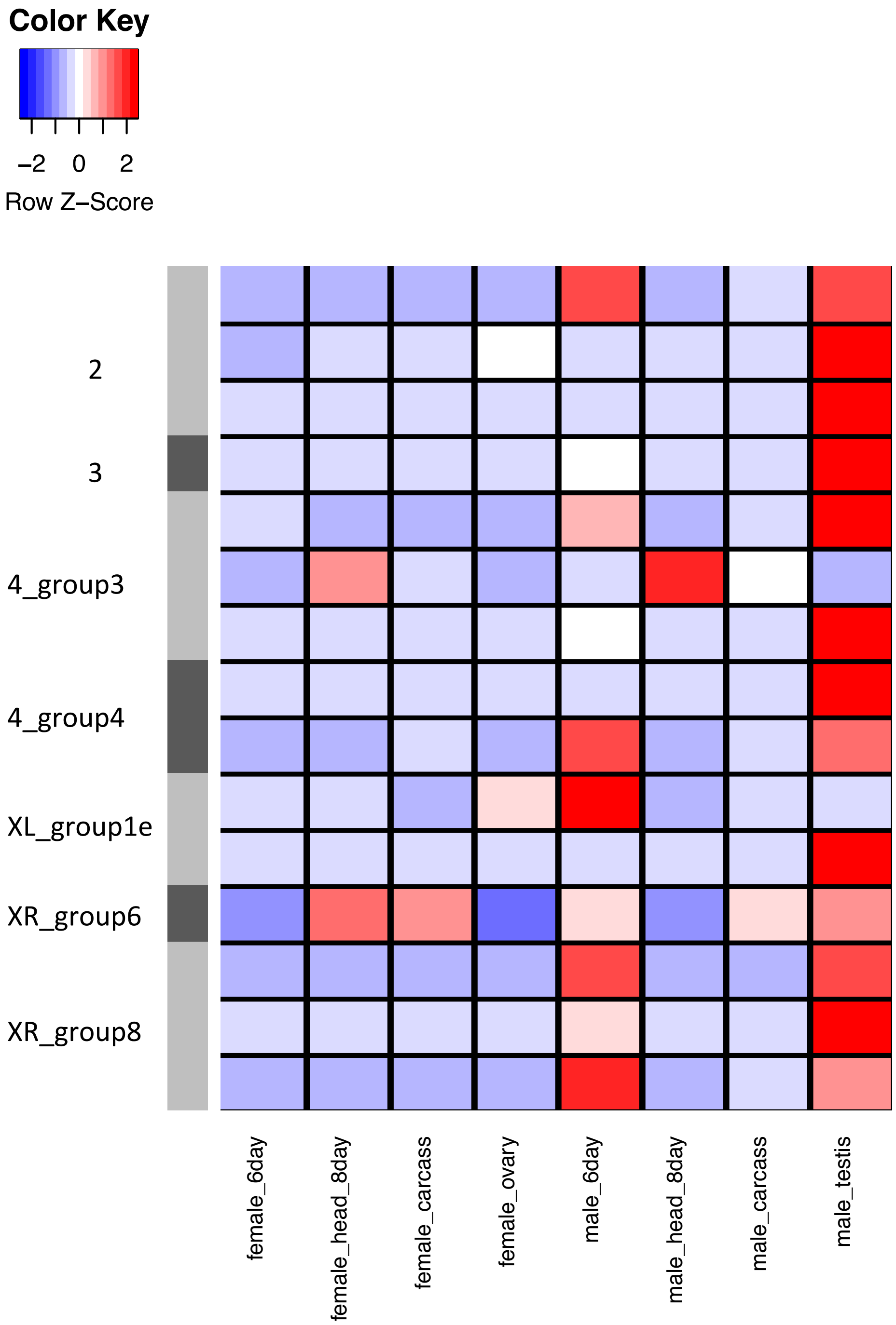

Supplementary Figure 7. Expression patterns of autosomal or X-linked paralogs of testis-expressed Y-linked transcripts in *D. pseudoobscura.* Most testis-expressed genes that were acquired or maintained on the neo-Y of *D. pseudoobscura* ancestrally have testis-biased expression (as inferred based on expression patterns of their paralogs). Each row corresponds to a gene, and genes are grouped by chromosomal location in *D. pseudoobscura.*

Supplementary Table 1. Overview of all datasets used for this study, including Genbank accession numbers.

species	genomic data		transcriptome data	
	male	female	male	female
Chironomus riparius	SRR1738174	SRR1738173	PRJNA385725*	PRJNA385725*
Chaoborus trivittatus	SRR1738213	SRR1738278	PRJNA385725*	PRJNA385725*
Anopheles gambiae	SRR1509742	SRR1508169	SRR535750	SRR953402
Aedes aegypti	SRR1738168	SRR1738167	SRR924021 (testis)	SRR1585315
Clogmia albipunctata	SRR1738153	SRR1738152	PRJNA385725*	PRJNA385725*
Tipula oleracea	SRR1738202	SRR1738201	PRJNA385725*	PRJNA385725*
Coboldia fuscipes	SRR1738157	SRR1738156	PRJNA385725*	PRJNA385725*
Mayetiola destructor	SRR1738190	SRR1738189	SRR1738673	SRR1738672
Condylostylus patibulatus	SRR1738159	SRR1738158	PRJNA385725*	PRJNA385725*
Megaselia abdita	SRR1738192	SRR1738191	PRJNA385725*	PRJNA385725*
Themira minor	SRR1700645, SRR1700634	SRR1700632, SRR1700633	SRR1700682	SRR1700646
Bactrocera oleae	SRR826808	SRR826807	PRJNA385725*	PRJNA385725*
Teleopsis dalmanni	SRR1738200	SRR1738199	SRR1738676	SRR1738677
Liriomyza trifoli	SRR1700531	SRR1700530	SRR1700443	SRR1699519
Ephydra hians	SRR1738182, SRR1738176	SRR1738181, SRR1738175	SRR1738666	SRR1738664
Phortica variegata	SRR826813	SRR826812	SRR1738675	SRR1738674
Drosophila pseudoobscura	SRR1738164	PRJNA385727*	SRR357403	SRR357405
Drosophila miranda	SRR1738163	SRR1738162	SRR364798	SRR364800
Drosophila melanogaster	SRR1738161	SRR1738160	SRR1197415	SRR1197317
Drosophila busckii	SRR826814	SRR826809	SRR1804796	SRR1805120
Drosophila albomicans	SRR1738314	SRR1738289	SRR402049	SRR402050
Sarcophaga bullata	SRR826794	SRR826793	PRJNA385725*	PRJNA385725*

* These data were newly collected for this study

Supplementary Table 2. Accession numbers of male and female genomic *D. melanogaster* reads used to study the newly identified Y-linked gene *CG41561*. Male and female strains were chosen at random from the NCBI SRA database.

SRA Accession	Sex	Strain	Source	Coverage (% of transcript)
ERR701706	Male	Iso1	Bloomington	Yes (75%)
ERR701712	Male	nos-GAL4; UAS-DCR2	Greg Hannon Lab	Yes (60%)
SRR1525699	Male	Wild caught	USA: Bowdoinham, ME, Paul Schmidt	Yes (84%)
SRR1525770	Male	Wild caught	USA: Linvilla, PA, Paul Schmidt	Yes (90%)
SRR1738161	Male	Canton-S Lab strain	Stock center	Yes (80%)
ERR705952	Female	Haddrill France 31	Isofemale line collected in Montpellier, FRANCE	None
ERR705984	Female	Haddrill Georgia Pool15	15 isofemale strains collected in Athens, Georgia	None
SRR2134629	Female	Canton-S	Mark Biggin and Eisen Lab, UC Berkeley	None
SRR492060	Female	Z30	Carnegie Mellon University, Joel McManus	None
SRR1738160	Female	Canton-S Lab strain	Stock center	None

Supplementary Table 3. Genome and transcriptome assembly statistics for Diptera species considered, and false-positive rate of our bioinformatics pipeline (as measured by the number of male- and female-specific transcripts for each taxon). Species are ordered as in ref. [1].

Species	Sex chromosome	Genome statistics					Male-specific Transcriptome statistics				Sex-specific contigs	
		N50 (bp)*	Total assembled genome size (Mb)	Number of raw sequencing reads pairs (x 10^6)	coverage	% reads mapping to assembled genome	Transcript N50 (bp)	median transcript length	average transcript length	Total size male-specific transcriptome	# male-specific contigs	# female-specific contigs
Chironomus riparius	homo	14,912	185.4	12.7	~12X	67.5	350	299	364.55	4,107,046	8	6
Chaoborus trivittatus	homo	2,743	408.9	31.5	~14X	47.3	320	287	334.1	6,158,470	17	2
Anopheles gambiae	XY	7,484	219.5	15.6	~13X	62.1	401	318	399.95	6,889,454	3	0
Aedes aegypti	homo	1,619	726.5	27.3	~7X	35.8	438	340	428.96	8,617,313	2	11
Clogmia albipunctata	homo	11,179	310.8	19.0	~11X	60.2	392	305	411.9	5,987,392	21	0
Tipula oleracea	XY	1,603	534.8	18.7	~6X	35.8	473	342	444.94	17,549,300	4	2
Coboldia fuscipes	XY	241,756	102.8	17.7	~31X	88.6	431	333	438.98	82,089	0	0
Mayetiola destructor	X0	14,122	154.5	7.7	~9X	69.5	349	302	356.28	7,117,333	0	0
Condylostylus patibulatus	XY	1,571	569.4	53.0	~17X	38.8	360	302	369.35	5,251,364	8	20
Megaselia abdita	homo	4,378	485.8	38.8	~14X	43.7	387	306	404.29	3,790,178	0	0
Themira minor	XY	2,212	114.9	16.9	~27X	67.8	355	301	366.09	3,861,476	16	1
Bactrocera oleae	XY	8,832	390.6	40.0	~18X	90.2	339	293	354.72	2,093,914	2	1
Teleopsis dalmanni	XY	4,268	575.0	39.2	~12X	69.9	470	341	449.72	8,185,400	22	2
Liriomyza trifolii	XY	1,633	125.9	19.3	~28X	45.5	363	307	370.54	10,049,396	1	0
Ephydra hians	XY	2,261	487.2	32.4	~12X	71.3	394	316	396.62	10,619,382	9	2
Phortica variegata	XY	37,343	253.2	13.0	~9X	54.8	1148	380	707.35	32,025,171	16	3
Drosophila pseudoobscura	XY	55,872	134.4	15.7	~21X	76.1	603	368	526.76	39,006,511	76	3
Drosophila miranda	XY	24,439	175.1	8.2	~8.5X	62.5	684	370	565.95	53,483,655	433	0
Drosophila melanogaster	XY	88,921	120.2	15.1	~23X	81.1	530	316	496.27	10,464,410	29	3
Drosophila busckii	XY	36,315	121.1	25.8	~38X	78.3	445	327	434.28	14,172,673	139	0
Drosophila albomicans	XY	35,303	158.2	12.0	~14X	68.4	348	298	360.53	6,173,786	61	0
Sarcophaga bullata	XY	1,828	484.6	38.0	~14X	66.4	381	298	388.54	1,242,166	0	4

*female-only genome assembly

Supplementary Table 4. PCR primers amplifying male-specific products in Diptera species (i.e. Y-linked transcripts).

species	transcript	for_primer	rev_primer	PCR product (bp)	notes
Themira minor	CL166Contig1	TCGACGTTGTGCTCTTTGAG	GAGCCACGTGAATGTTCAGA	225	protein-coding
Themira minor	TR7942	CAGTCGGATGACTTCGTTCC	TTTGAATTTTCGTCGTGTGC	186	
Themira minor	TR242	TGTCCTCCTTTGGGTTTCAC	ACCGCAAGTTTCTCGGAATA	175	
Themira minor	TR3320	AGAGCGTCCTTCCTTTGTGA	CGATGACGGTGATCTTGTTG	195	
Themira minor	CL55Contig1	AGTTTCGGACAAGAGCAGGA	AATATCCGTTTGGTGCTTGC	199	
Themira minor	CL11Contig2	GGCCTTCGGATTTTAGGAAG	AAGACTACTTGCGCGACGAT	186	
Teleopsis dalmanni	TR12456	TTTGGCGTTATGTTCCTGGT	CCGCTATTTTCCCCCATAGT	237	protein-coding
Teleopsis dalmanni	TR8004	CATCAAAGAGTGGCACAGGA	AACCTTTTGCTGCGCTTCTA	164	protein-coding
Teleopsis dalmanni	TR2473	TTGCCTGGAAAAAGAATTGG	TGTTAGCTTCATGCGAAACG	182	protein-coding
Teleopsis dalmanni	TR4930	ACAGGCTCGCTAAGTTGGAA	ACCCTAACGGATCCACACAA	225	protein-coding
Teleopsis dalmanni	CL466Contig1	CGATTACCGATTGCTCCATT	AGGCACTACCGATAGCGAGA	192	protein-coding
Teleopsis dalmanni	TR6579	CGTATGTCTCGCCGAGTGTA	ACAAGCGCTTCAGATCTTCC	151	protein-coding
Teleopsis dalmanni	TR2554	TTTACAGTTCCCTCGGATGC	TGCATCATTTGAAAGGGATTT	195	protein-coding
Teleopsis dalmanni	CL291Contig1	TCGGGAATAACGCAGGATAC	CTTGGGGCATCATTTTGTTT	218	protein-coding
Teleopsis dalmanni	TR6646	TCACCAATGCACCAACATTC	CGTTGCAAATAAGCATCCAA	209	
Teleopsis dalmanni	TR8935	TCCATTTGGAGTGGACCTGT	GCACATGCTTCGAATTGTTG	154	
Ephydra hians	CL425Contig1	CGTACCAGAGCAGACACCAA	AACCAAACAAACAGAGCTTGC	195	
Ephydra hians	CL517Contig1	TGAAGAACTTTGTTGGTGCATT	CCGTCCGGAATGTGTTTATG	249	
Ephydra hians	CL517Contig1*	ACGGGAGGAAATTCGAGTAA	TTGGTGTCTGCTCTGGTACG	246	
Ephydra hians	CL624Contig1	AAATAAAGCCGTTGAGCAGT	AACCTTTGCAAAACGCTATC		
Ephydra hians	CL624Contig1*	ATTATTTCCGTCGCACTTTC	TTTGAGCACAGTCACCAAAC		
Ephydra hians	TR1001	CTTTGATCTTGGCCGTGTTT	AAGAACTTTTCTGATGCATTGCT	213	
Ephydra hians	TR10871	GCATCAGTAAAAGGGGCAAA	GACGCCTAAGGCCATCATTA	160	protein-coding
Ephydra hians	TR18873	CAAAGGGTGCGTCCATTATC	ACAGCGTTTTGTCTCCGTTT	186	
Phortica variegata	CL1175Contig1	CCTATTGCAGCTGATGACCA	TGGCACAAGTTTCAGCAGAC	295	protein coding
Phortica variegata	CL542Contig1	GTCGCCAATGTGACTCTGAA	CTGCTGTTTTCAGCCATCAA	299	protein coding
Phortica variegata	TR15734	TCTCCCTGAATTTACCAAAGGA	TTTGCGGTTCGGAAAAATTA	293	
Phortica variegata	TR18029	TTTAACAAATTCGCGGTCAT	TGCATTATAAGATGGCAGGA	97	
Phortica variegata	TR18029*	TTACAGCGGTACAACCAAAA	GACAACTCAGATTCGGACATT	101	
Phortica variegata	TR25433	TTGGGTCATGCATAGAGAAA	AGAACTTGTGGGAGAATGGA	100	
Phortica variegata	TR25433*	ATCCAAGACTGGAGGTTCAA	GCCAGATCGCATTTCTCTAT	81	
Phortica variegata	TR25932	CGTCTCCTCCTCATCTTTGC	TCACCGTTTCTCACAGAGCA	278	
Phortica variegata	TR28140	ATTCGTTCAGGCTCATTTGT	GCCACGTTTGATCCAATACT	77	
Phortica variegata	TR28140*	GCACAGAAAAACCGGAGTAT	TGTTTTGCCTTCTATGTGCT	83	
Phortica variegata	TR6288	AAATGTGGGAGAATTGGAAA	GAACTAAACTGGGGAGCTGA	299	
Phortica variegata	TR6288*	TATTCCAACCCCTGGTAAAA	AAGGCGTGTATTTCCTTCTG	307	

Supplementary Table 5. Accession numbers of tissue-specific expression data.

species	Somatic tissues		Gonads	
	Male head	Female head	Testis	Ovary
Themira minor	SRR1700709	SRR1700675	SRR1700693	SRR1700647
Teleopsis dalmanni	SRR1184533	SRR1184534	SRR1184544	SRR1184546
Ephydra hians	SRR1738668	SRR1738667	SRR1738671	SRR1738669
Drosophila pseudoobscura	SRP001791	SRP001791	SRR357404	SRR357400
Drosophila miranda	SRR4416188	SRR4416186	SRR364799	SRR364801
Drosophila melanogaster	SRR070400, SRR070416	SRR070430, SRR100282	SRR100276, SRR070422	SRR070396, SRR070417
Drosophila albomicans	SRR4416171	SRR4416190	SRR4416172	SRR4416191

Supplementary References

1. Vicoso, B. & Bachtrog, D. Numerous transitions of sex chromosomes in Diptera. *PLoS Biol.* **13,** e1002078 (2015).
